# Neglected, yet significant role of FOXP1 in T-cell quiescence, differentiation and exhaustion

**DOI:** 10.3389/fimmu.2022.971045

**Published:** 2022-10-04

**Authors:** Yaroslav Kaminskiy, Varvara Kuznetsova, Anna Kudriaeva, Ekaterina Zmievskaya, Emil Bulatov

**Affiliations:** ^1^ Department of Oncology and Pathology, Karolinska Institutet, SciLifeLab, Solna, Sweden; ^2^ Laboratory of Transplantation Immunology, National Research Centre for Hematology, Moscow, Russia; ^3^ Shemyakin-Ovchinnikov Institute of Bioorganic Chemistry, Russian Academy of Sciences, Moscow, Russia; ^4^ Institute of Fundamental Medicine and Biology, Kazan Federal University, Kazan, Russia

**Keywords:** FoxP1, T cell, Treg, quiescence, T cell differentiation, isoforms, antitumor, exhaustion

## Abstract

FOXP1 is ubiquitously expressed in the human body and is implicated in both physiological and pathological processes including cancer. However, despite its importance the role of FOXP1 in T-cells has not been extensively studied. Although relatively few phenotypic and mechanistic details are available, FOXP1 role in T-cell quiescence and differentiation of CD4+ subsets has recently been established. FOXP1 prevents spontaneous T-cell activation, preserves memory potential, and regulates the development of follicular helper and regulatory T-cells. Moreover, there is growing evidence that FOXP1 also regulates T-cell exhaustion. Altogether this makes FOXP1 a crucial and highly undervalued regulator of T-cell homeostasis. In this review, we discuss the biology of FOXP1 with a focus on discoveries made in T-cells in recent years.

## FOXP1 biology overview

The gene encoding forkhead box protein P1 (*FOXP1)* was originally discovered and cloned in 2001 (1, 2). The full-length FOXP1 protein is 677 amino acids long, and its gene is located in 3p14.1 chromosomal region. FOXP1 is ubiquitously expressed in normal tissues and belongs to the family of forkhead box (FOX) proteins, which all share a conserved DNA-binding domain (winged helix) (3). In addition, FOXP1 is a member of a smaller FOXP subgroup, which includes FOXP1-4 and has leucine zipper and zinc finger domains (4). FOXP1 can form homo- and heterodimers with itself and other members of the FOXP family through the leucine zipper motif, and the dimerization is necessary for the transcriptional activity (5–7). Further, it can interact with CtBP-1:NuRD complex and generally acts as a transcriptional repressor (8, 9).

Alternative splicing of FOXP1 can generate 7 isoforms (total number of predicted isoforms is 23) in human and 23 isoforms in murine cells (10, 11). FOXP1 DNA-binding site is highly conserved (90% homology) between humans and mice (10). Besides, human FOXP1 isoforms 3 and 4 share 50% homology to murine isoforms 2, 3, 4 and 5.

FOXP1 analysis in neurological disorders identified 7 mutations in the protein (A339Sfs*4, V423Hfs*37, Y439*, R465G, R514C, R525*, W534R) (12, 13). All of them disrupted the transcriptional activity of FOXP1, whereas three also disrupted FOXP1 ability to homo- and heterodimerize (A339Sfs*4, R525*, W534R). V423Hfs*37, R465G, and R514C variants, on the other hand, bound and sequestered wild type FOXP1 and FOXP2, hence, exerting a dominant-negative effect. ChIP-seq analysis of murine CD4+ T-cells revealed 3071 FOXP1-bound sites in the Treg subset and 1088 sites in the naïve subset (14). Interestingly, FOXP1 *de novo* motif discovery performed by the same group showed that only 17% of binding sites contained canonical forkhead motif, (G/A)T(A/C)AA(C/T)A. Whereas, over 26% of binding sites contained ETS motif, GGA(A/T). Relatively low occurrence of forkhead motif in FOXP1 binding sites suggests the important role of other binding partners.

FOXP1 is implicated in various physiological settings including B-cell development, monocyte differentiation, and lung epithelia regeneration (15–17). In cancer it can act as both an oncogene (B-cell lymphoma, ovarian cancer, hepatocellular carcinoma) and a tumor suppressor (T-cell lymphoma, NSCLC, colorectal cancer) (18–22). Moreover, there is evidence that in breast cancer cells, FOXP1 expression dampens the synthesis of T-cell attracting cytokines and, as a result, impairs T-cell infiltration (23).

Further in the first section we review the molecular biology of FOXP1 in human CD4+ T-cells and then in the second section we discuss FOXP1 role in T-cell quiescence and exhaustion. In the third section we have a closer look at FOXP1 in the context of CD4+ T-cell differentiation. Finally, in the fourth section we briefly cover discrepancies among various isoforms.

## FOXP1 biology in human CD4+ T-cells

Durek et al. (2016) was the first to look at FOXP1 role in human CD4+ T-cells and found 4 isoforms of *FOXP1* in their RNA-seq dataset (24). 3 of those isoforms contained complete protein coding sequences, whereas the 4^th^ isoform contained only 3 exons. Expression levels of all detected *FOXP1* isoforms were the highest in the naïve (Tn) subset, substantially decreased in the central memory (Tcm) subset and the lowest in the effector memory (Tem) subset. This expression downregulation was accompanied by the increased DNA methylation in *FOXP1* promoter suggesting epigenetic shut-down of FOXP1 in more differentiated CD4+ T-cell subsets.

Garaud et al. (2017) performed another comprehensive investigation of FOXP1 biology in human CD4+ T-cells (25). They identified that 6 out of 23 predicted FOXP1 splice isoforms are expressed in human PBMCs and CD4+ T-cells and concluded that the full-length isoform was the most abundant one. In agreement with Durek et al. (2016), FOXP1 expression in CD45RA+CD4+ T-cells (mostly naïve) was greater than in CD45RA-CD4+ subpopulation (memory). Also, in line with Durek et al. (2016), memory T-cells showed increased DNA methylation in *FOXP1* promoter. FOXP1 RNA and protein levels were shown to decrease in naïve and memory CD4+ T-cells upon T-cell receptor (TCR) stimulation. Bypassing TCR stimulation and activation of PKC-mediated Ca^2+^ influx directly did not achieve the same level of FOXP1 repression, suggesting that other TCR pathways play a role in FOXP1 downregulation. Interestingly, the downregulation in naïve T-cells was more pronounced than in memory T-cells. Another interesting observation by the authors is that the expression of a shorter isoform (65 kDa) increased 24 hours after activation but then decreased back to initial levels. FOXP1 repression by shRNA increased the number of cycling and activated cells but did not affect apoptosis. The authors performed FOXP1 ChIP-qPCR analysis and found that FOXP1 bound and repressed *ID2, STAT6, IL-13*, and *IL-17A* in naïve T-cells, but only *ID2* in memory T-cells. The products of these genes play role in T-cell differentiation and effector functions, suggesting that FOXP1 can preserve stemness in T-cells (26). Importantly, FOXP1 did not bind *FOXP3* promoter but *FOXP3* expression was increased in *FOXP1*-knockdown memory T-cells but not naïve CD4+ T-cells, indicating that FOXP1 can repress *FOXP3* expression.

Although the aforementioned studies elucidated many aspects of FOXP1 in human CD4+ T-cells, additional studies are needed to investigate FOXP1 in the context of CD4+ T-cell memory formation, antitumor function, and autoimmunity.

## FOXP1 role in T-cell quiescence

Early evidence of FOXP1 role in T-cells came from a study of lymphocytic variant of hypereosinophilic syndrome (L-HES) patients. L-HES is a benign lymphoproliferative disease that is characterized by clonally expanded CD3-CD4+ T-cells and, in some cases, can progress to T-cell lymphoma (27). Expansion of these abnormal Th2 cells and their IL-5 secretion increase eosinophil counts. Gene expression profile of abnormal T-cells was compared to that of normal CD3+CD4+ T-cells (28). This analysis showed significant downregulation of FOXP1 in abnormal T-cells suggesting a potential role in T-cell quiescence. Indeed, in patients with peripheral T-cell lymphoma *FOXP1* expression inversely correlated with proliferation marker Ki-67 (19). FOXP1 overexpressing tumors were associated with better survival. Surprisingly, this was not the case for B-cell lymphomas, which suggests different role of FOXP1 in these cell types (29, 30). Another explanation of this discrepancy is preferential expression of N-terminal truncated FOXP1 isoforms (60 and 65 kDa) by malignant B-cells, which seem to have an oncogenic role (31, 32). However, another report showed that, although short FOXP1 isoform is preferentially expressed in B-cell lymphomas, overexpressed full-length FOXP1 has a similar oncogenic activity (33). Feng et al. (2010) used a mouse model with conditional deletion of *FOXP1* at CD4+CD8+ stage of thymocyte development and showed that both mature *FOXP1-/-* CD4+ and CD8+ T-cells lost their quiescence (34). These cells had larger size, downregulated CD62L, upregulated CD44, and were more prone to IFN-γ production and apoptosis. Heterozygous *FOXP1* deletion led to more activated phenotype only in CD8+ T-cells, suggesting that CD4+ T-cells are less sensitive to the changes in *FOXP1* levels. The authors did not observe any defects in Tregs formation and function. Similar activated CD62L-CD44+ phenotype in CD4+ cells was observed by an independent group in a mouse model with conditional *FOXP1* deletion (24). In their subsequent study, Feng et al. (2011) used tamoxifen-mediated deletion of *FOXP1* in mature T-cells (35). They showed that FOXP1 directly repressed IL-7Ra expression and, thus, prevented IL-7-driven antigen-independent proliferation of naïve T-cells *in vitro* and *in vivo*. Upon *FOXP1* deletion, both naïve CD8+ and CD4+ subpopulations demonstrated activated phenotype but only CD8+ proliferated in response to IL-7. FOXP1 was shown to inhibit MEK/Erk pathway activation independently from IL-7Ra expression. They also confirmed that complete (homozygous) deletion of *FOXP1* in CD8+ T-cells was necessary for the change in phenotype. In summary, it was shown that in CD8+ T-cells FOXP1 induces quiescence through downregulation of MAPK pathway and responsiveness to IL-7. Interestingly, FOXP1 protein bound the same enhancer as FOXO1, antagonizing IL-7Ra expression activation by FOXO1. *FOXO1* deletion can also lead to spontaneous T-cell activation, but FOXO1 is necessary for IL-7Ra expression (36). Given FOXO1 role in the restriction of T-cell effector differentiation and in memory formation, it seems that FOXP1 is more important for the naïve T-cell quiescence (37). On the other hand, memory T-cells have reduced FOXP1 and increased FOXO1 expression, which potentially contributes to homeostatic proliferation. In another study, this group elucidated the mechanistic details of FOXP1-induced T-cell quiescence (38). They used the same conditional *FOXP1* deletion in mature CD8+ T-cells to demonstrate that *FOXP1* deficient cells activated PI3K/Akt/mTOR pathway in response to IL-7 and that FOXP1 is necessary for the expression of PI3K inhibitor, Pik3ip1. They also showed that *FOXP1* deficient cells had higher expression of cell-cycle E2F factors and their target genes (*MCM5*, *CDK1*, *PCNA*).

**Figure 1:**
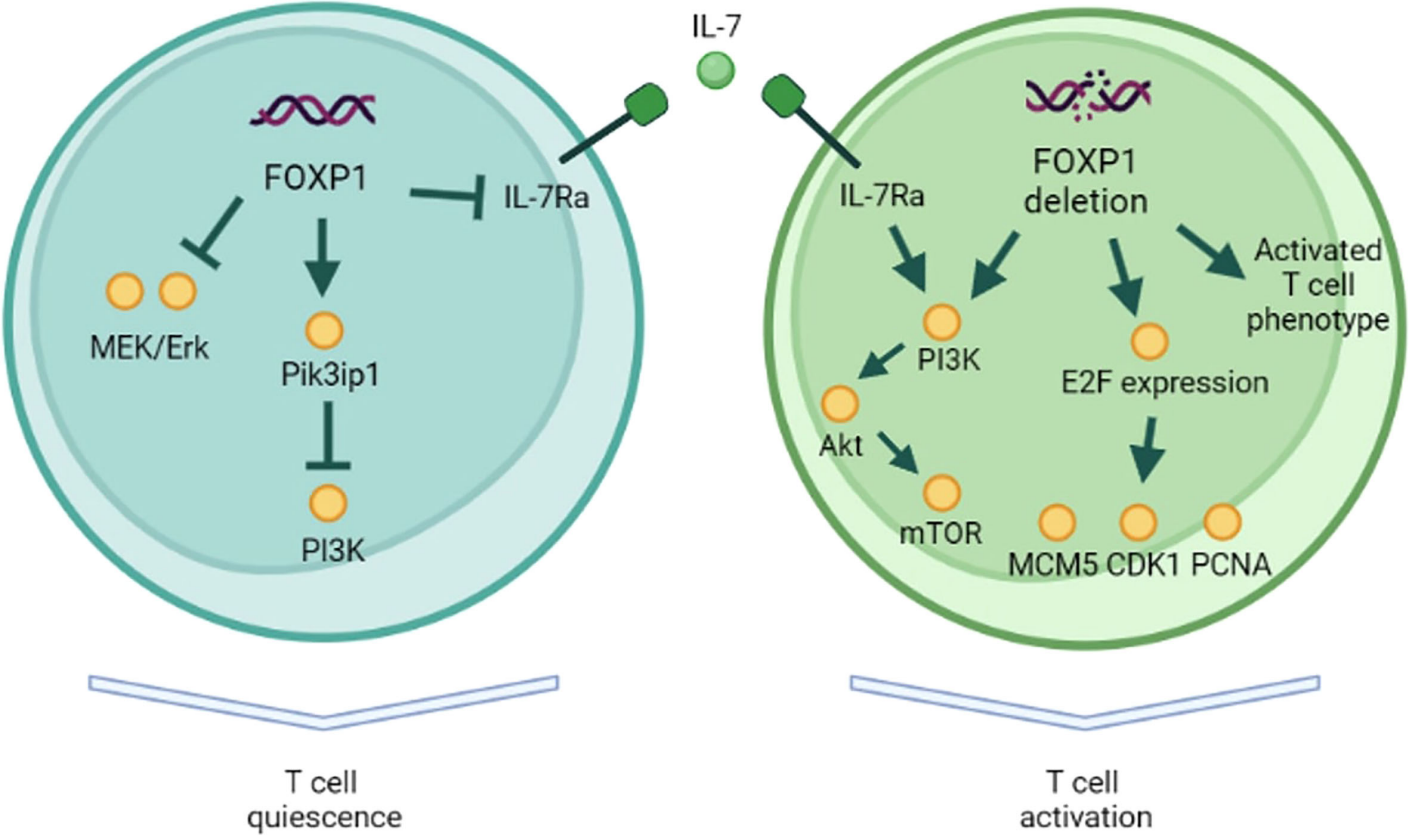
FOXP1 role in T-cell quiescence. In naïve T-cells FOXP1 inhibits MAPK and PI3K pathways. FOXP1 also represses IL-7Ra expression, preventing IL-7-driven antigen-independent proliferation. FOXP1-deficient naïve T-cells demonstrate activated surface phenotype, PI3K pathway activation in response to IL-7, and the higher expression of E2F factors and their target genes (*MCM5*, *CDK1*, *PCNA*).

Although FOXP1 biology in T-cells is only beginning to reveal its secrets, there is evidence to claim FOXP1 role in maintaining T-cell quiescence in mice (**Figure 1**). T-cell quiescence is essential not only for preventing spontaneous activation and autoimmunity but also for maintaining a pool of naïve and memory T-cells that can mount immune response. The constantly proliferating T-cells are expected eventually reach Hayflick limit and lose their ability to expand further, whereas FOXP1 counteracts this process and preserves T-cell expansion capacity.

## FOXP1 role in T-cell exhaustion and antitumor immunity

The FOXP1-induced quiescence in T-cells was shown to have implications for the antitumor immunity. Stephen et al. (2014) analyzed FOXP1 in the context of TGF-β signaling in the tumor microenvironment (39). They showed that human CD8+ T-cells expressed both FOXP1A (full-length) and FOXP1D. FOXP1A was downregulated upon activation, whereas both isoforms were upregulated in tumor-infiltrating lymphocytes. IL-2, IL-6, IL-7, IL-15, IL-17, IL-23 and VEGF-α did not affect FOXP1 expression, while ICAM1, CXCL12, and TGF-β signaling induced FOXP1 upregulation. Moreover, FOXP1 interacted with SMAD2/3, and this interaction was required for TGF-β repressive activity. Accordingly, FOXP1 deficiency improved antitumor immunity in ovarian cancer and sarcoma models. In agreement with this work, another group showed that the TGF-β pathway activity as well as FOXP1 expression in human CD8+ T-cells were elevated after co-culture with M2 inhibitory macrophages (40). As TGF-β signaling is implicated in T-cell exhaustion, it is reasonable to assume that FOXP1 (possibly in cooperation with SMAD2/3) also regulates this dysfunction state in T-cells. Similarly to human TILs, *FOXP1* expression was higher in CD8+ T-cells from a tumor-bearing mouse supporting its role in T-cell exhaustion (41). Overexpression of miR-149-3p which inhibits PD-1, TIM-3, BTLA, and FOXP1 translation, counteracted CD8+ T-cell exhaustion. Although additional work is needed to expand our knowledge, there is already growing evidence that FOXP1 may play an important role in T-cell exhaustion, affecting T-cell ability to fight tumor.

## FOXP1 role in CD4+ T-cell differentiation

In addition to the abovementioned functions FOXP1 also regulates differentiation of CD4+ T-cells. Wang et al. (2014) showed that in the murine system FOXP1 suppresses differentiation of CD4+ T follicular helper cells (Tfh) (42). Tfh cells facilitate B-cell maturation and germinal centers formation, and also express high levels of Bcl-6, CXCR5, ICOS and IL-21 (43–46). Contrary to what has been shown by Garaud et al. (2017) in human CD4+ T-cells, the expression of full-length FOXP1 isoform (FOXP1A) in mouse CD4+ T-cells did not change upon TCR stimulation, whereas the levels of another isoform (FOXP1D) increased both *in vitro* and *in vivo*. Because both FOXP1A and FOXP1D can repress Tfh, this increase in total FOXP1 levels led to indirectly reduced expression of ICOS, which is necessary for Tfh differentiation. The authors hypothesized that lower ICOS expression was due to FOXP1 inhibitory effect on MEK/Erk signaling that was shown to induce ICOS expression (47). FOXP1 also directly repressed the expression of IL-21, which is important for both Tfh differentiation and B-cell maintenance. Later this group demonstrated that FOXP1 directly regulates CTLA4, which is important for Tfh differentiation and function (48). They also showed that FOXP1-deficient CD4+ T-cells downregulated CCR7 receptor and, hence, migrated to B-cell follicles earlier during the infection. Similarly, FOXP1 was also demonstrated to negatively regulate Th9 differentiation (49). Th9 CD4+ T-cell subset is characterized by IL-9 and IL-21 secretion and plays a role in antitumor immunity (50–52). The authors showed that in mouse CD4+ T-cells FOXP1 directly repressed IL-9 expression by binding to its promoter. FOXO1, on the other hand, induced IL-9 expression and antagonized FOXP1 at IL-9 promoter. It is noteworthy that IL-7 signaling was responsible for FOXP1 nuclear exclusion and FOXO1 nuclear localization. Interestingly, the expression of full-length FOXP1 isoform (FOXP1A) increased after murine CD4+ T-cell activation, which contradicts the abovementioned results of Wang et al. (2014).

FOXP1 plays an important role in homeostasis of regulatory T-cells. In inducible Tregs (iTregs) FOXP1 was essential for both the proper differentiation and the stability of mature Treg lineage (53). Mechanistically, upon TGF-β treatment, FOXP1 directly bound *FOXP3* promoter and enhancer, and this interaction was associated with an increase in permissive chromatin modifications (H3K4me3, H3K9/K14Ac), sustaining *FOXP3* expression. On the other hand, in thymus Tregs (tTregs) FOXP3 expression was not affected by *FOXP1* deletion, and this cell population was stable in the absence of FOXP1. Dependence of iTregs on FOXP1-driven *FOXP3* expression was confirmed by another group (54). They demonstrated that, as opposed to naïve murine CD4+ cells, activated Tregs downregulated FOXP1A expression similar to human CD4+ T-cells. They also showed that *FOXP1* deletion led to increased number of activated Tregs with impaired suppressive function and that genes downregulated in *FOXP1* deficient cells were significantly enriched in TGF-β pathway. FOXP1 was shown to increase FOXP3 recruitment to target genes and regulate *CTLA4* expression through FOXP1-FOXP3 axis (14). FOXP1 and FOXP3 shared the majority of promoter binding sites and regulated target genes expression largely in a cooperative manner. FOXP1 deficiency reduced FOXP3 DNA binding ability and led to decreased CD25 expression. Interestingly, some features of Treg differentiation in FOXP1-deficient cells were rescued by stronger IL-2 signaling, which suggests an existence of compensatory mechanisms.

**Figure 2:**
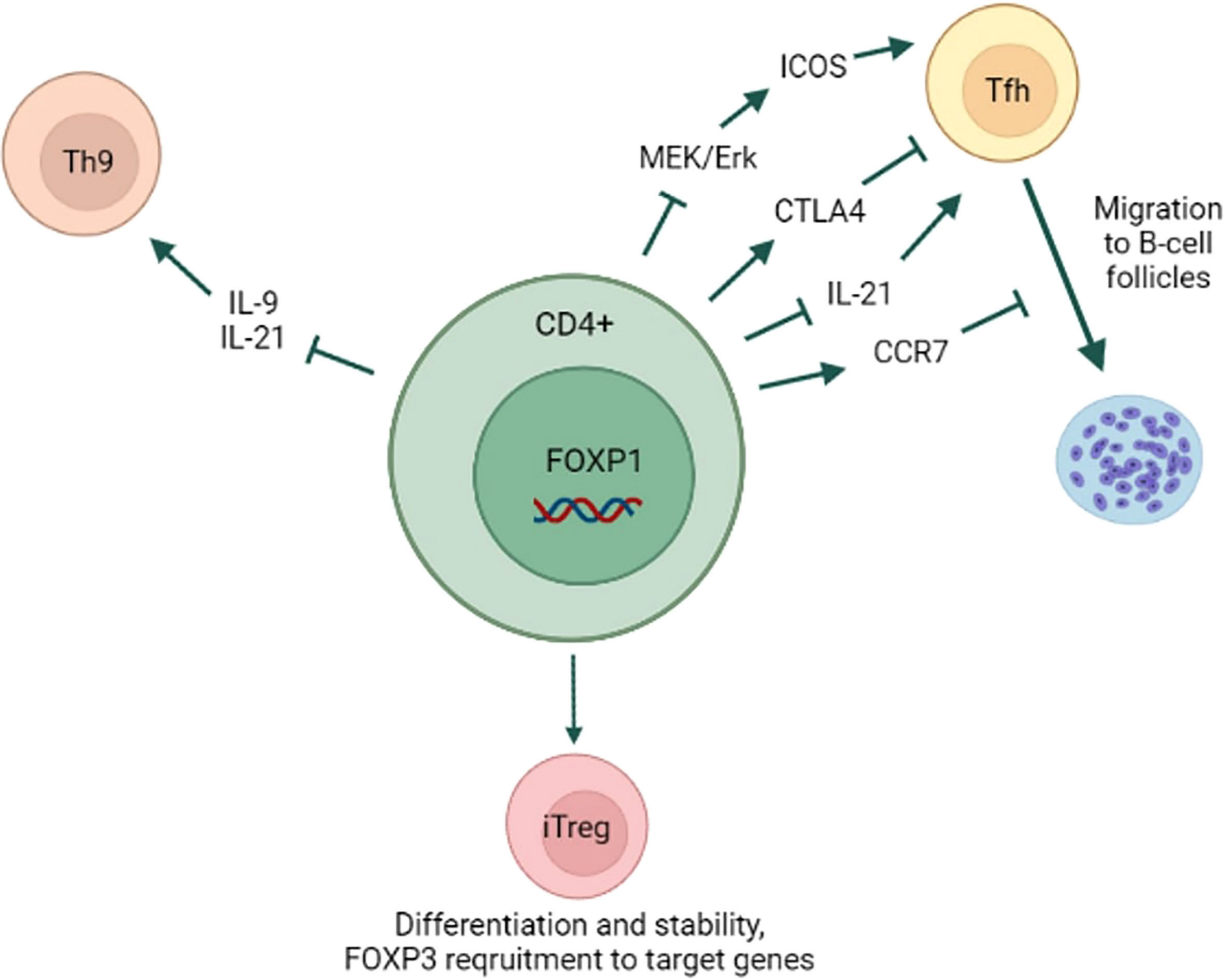
FOXP1 role in CD4+ T-cell differentiation. FOXP1 prevents naïve CD4+ T-cell differentiation into Tfh subset by inhibiting MAPK/ICOS axis and IL-21 expression. FOXP1 promotes CTLA4 and CCR7 expression, which inhibit Tfh formation and the migration to B-cell follicles, respectively. By repressing IL-9 and IL-21 expression FOXP1 prevents CD4+ T-cell differentiation into Th9 subset. FOXP1 is essential for iTregs differentiation and lineage stability likely through FOXP3 recruitment to its target genes. Yet, FOXP1 is dispensable for thymus Tregs differentiation and lineage stability.

Summarizing all the above, FOXP1 plays an important role in Tfh and Treg differentiation in mouse CD4+ T-cells (**Figure 2**). Its role in other CD4+ T-cell subsets has not been studied extensively so far. Moreover, further studies are needed to confirm whether FOXP1 also regulates differentiation of human CD4+ T-cells.

## Confusion with FOXP1 isoforms

It is noteworthy that a discrepancy exists in FOXP1 expression kinetics after T-cell activation (**Table 1**). Some reports demonstrated FOXP1 downregulation, while others showed the opposite effect (25, 39, 42). TCR activation presumably induces expression of short FOXP1 isoform that can act as a dominant-negative regulator with respect to full-length FOXP1 (25). The full-length FOXP1 preserves the quiescent state, therefore it should be inhibited during antigen-driven activation and expansion (34–38). However, both isoforms (full-length FOXP1 and truncated FOXP1D) share certain functionality given that either can repress Tfh differentiation (42). Similar to T-cells, B-cells also upregulate short FOXP1 isoforms upon activation that can act as oncogenes in B-cell malignancies (31, 32).

**Table 1:** FOXP1 isoforms expression after T-cell activation.

FOXP1 isoforms	Human CD8+	Human CD4+	Murine CD4+
FOXP1A (full-length)	downregulated; upregulated in TILs	downregulated	upregulated or no change; downregulated in iTregs
FOXP1D	unclear; upregulated in TILs	upregulated within 24h after activation; downregulated beyond 24h after activation	upregulated

It is essential to clarify which FOXP1 isoforms are expressed in human and mouse T-cells and how conserved they are between the species. This will remove an ambiguity that is currently present around FOXP1 isoforms.

## Conclusion

In the light of recent evidence, it would be safe to say that FOXP1 is one of the essential intrinsic factors that keep mouse CD8+ T-cells in quiescent state. In human CD8+ T-cells, FOXP1 modulates TGF-β signaling transduction but its role in quiescence is yet to be validated. In mouse CD4+ T-cells FOXP1 plays a role in Tfh and Treg differentiation. However, FOXP1 may not be as critical in murine thymus Tregs as in Tregs of a different origin. Given the extensive cooperativity between FOXP1 and lineage defining FOXP3, this may point to fundamental differences between Treg subsets. Intriguingly, unlike in mice, human CD4+ T-cells FOXP1 may repress FOXP3 expression, and this discrepancy should be carefully addressed by the future studies.

Although FOXP1 is not a therapeutic target as of now, it can potentially be manipulated for the therapeutic benefit especially if its connection to T-cell exhaustion is further validated. For example, *FOXP1* knockout in CAR-T-cells may improve their expansion and persistence. Alternatively, through the inhibition of Tfh differentiation, the reduction of FOXP1 levels may help alleviate autoimmune diseases driven by abnormal B-cell responses. Nevertheless, any therapeutic manipulation of FOXP1 mostly remains contingent on the future studies of its biology.

## Author contributions

YK and VK conceived the idea and wrote the manuscript. AK assisted with finalizing the manuscript. EZ prepared the figures. EB proofread the manuscript. All authors contributed to the article and approved the submitted version.

## Funding

This research was funded by the Ministry of Science and Higher Education of the Russian Federation (grant agreement no. 075-15-2020-795, state contract no. 13.1902.21.0027 of 29.09.2020 unique project ID: RF-190220X0027). 

## Acknowledgments

The work was performed in frames of Kazan Federal University Strategic Academic Leadership Program (PRIORITY-2030).

## Conflict of interest

The authors declare that the research was conducted in the absence of any commercial or financial relationships that could be construed as a potential conflict of interest.

## Publisher’s note

All claims expressed in this article are solely those of the authors and do not necessarily represent those of their affiliated organizations, or those of the publisher, the editors and the reviewers. Any product that may be evaluated in this article, or claim that may be made by its manufacturer, is not guaranteed or endorsed by the publisher.

## Abbreviations

ChIP-seq, Chromatin immunoprecipitation (ChIP); with DNA sequencing. FOXO1, Forkhead box protein O1; FOXP1, Forkhead box protein P1; FOXP3, Forkhead box protein P3; ICOS, Inducible T-cell costimulatory; IFN-γ, Interferon gamma; NSCLC, Non-Small Cell Lung Cancer; PKC, Protein kinase C; TGF-β, Transforming growth factor beta; Tfh, T follicular helper cells; Tn, naïve T-cells; Tcm, central memory T-cells; Tem, effector memory T-cells; Tregs, Regulatory T-cells.
